# Supplementary material for: The Impact of Liquid Biopsies Positive for EGFR Mutations on Overall Survival in Non-Small Cell Lung Cancer Patients
Source: Diagnostics (Basel). 2023 Jul 12;13(14):2347. doi: 10.3390/diagnostics13142347 (PMC10377956; doi:10.3390/diagnostics13142347)
Supplement: Supplementary file 1 [file diagnostics-13-02347-s001.zip › diagnostics-2378715-supplementary.pdf]

Supplementary Table 1. Correlation between the mutational status of the EGFR gene detected by liquid biopsy techniques and alterations in the *ALK, ROS1* and *BRAF* genes.

**Molecular Characteristics**	**Number of patients [n (%)]** *EGFR* **mutations detected by liquid biopsy**		Total of cases (n=38)	*P*
	**No (n = 11)**	**Yes (n = 27)**		
*ALK**				
Negative	8 (100)	24 (100)	32 (100)	NS
Positive	0 (0)	0 (0)	0 (0)	
*ROS1**				
Negative	1 (100)	5 (100)	6 (100)	NS
Positive	0 (0)	0 (0)	0 (0)	
*BRAF**				
Negative	2 (100)	3 (100)	5 (100)	NS
Positive	0 (0)	0 (0)	0 (0)	

The study of driver mutations in biopsy sample has been carried out according to current evidence at the time of diagnosis, but due to the scarcity of tumor tissue, alterations in the *ALK, ROS1* and *BRAF* genes were performed in 32, 16 and 5 patients; respectively.

Supplementary Table 2. Type of mutations detected in the *EGFR* gene in plasma samples from *EGFR* mutated non-small cell lung cancer (NSCLC) patients (*n*=38) during disease follow-up.

ID	Biopsy mutation	Number of liquid biopsies per patient						
		1	2	3	4	5	6	7
1	del(19)	WT	WT	WT	WT	del(19)	-	-
2	L858R	L858R	-	-	-	-	-	-
3	G719X+S578I	WT	WT	WT	WT	WT	WT	-
4	ins(20)	WT	-	-	-	-	-	-
5	L858R	L858R	-	-	-	-	-	-
6	ins(20)	ins(20)	-	-	-	-	-	-
7	L858R	WT	WT	WT	L858R	L858R	L858R	-
8	L858R	WT	WT	WT	WT	-	-	-
9	ins(20)	WT	-	-	-	-	-	-
10	del(19)	WT	del(19)	WT	-	-	-	-
11	L858R	L858R	-	-	-	-	-	-
12	del(19)	del(19)	WT	-	-	-	-	-
13	del(19)	del(19)	-	-	-	-	-	-
14	del(19)	WT	del(19)	WT	del(19)	del(19)	-	-
15	del(19)	WT	WT	WT	-	-	-	-
16	del(19)	L858R	-	-	-	-	-	-
17	L858R	WT	-	-	-	-	-	-
18	L858R	WT	WT	WT	WT	WT	WT	WT
19	L858R	L858R	L858R	-	-	-	-	-
20	del(19)	WT	-	-	-	-	-	-
21	L858R	WT	WT	WT	WT	-	-	-
22	L858R	L858R	WT	WT	L858R	-	-	-
23	L858R	WT	WT	WT	-	-	-	-
24	L858R	L858R	L858R	-	-	-	-	-
25	L858R	WT	-	-	-	-	-	-
26	L858R	L858R	L858R	-	-	-	-	-
27	del(19)	del(19)	del(19)+T790M	del(19)	del(19)	-	-	-
28	del(19)	del(19)	del(19)+T790M	-	-	-	-	-
29	L858R	L858R	L858R	WT	L858R+T790M	WT	WT	WT
30	del(19)	WT	del(19)+T790 M	-	-	-	-	-
31	del(19)+L861Q	del(19)+L861Q	del(19)+L861Q+T790M	-	-	-	-	-
32	del(19)+T790M	del(19)+T790M	del(19)+T790M	del(19)+T790M	-	-	-	-
33	del(19)	del(19)	del(19)	del(19)+T790M	-	-	-	-
34	L858R	L858R	WT	L858R+T790M	-	-	-	-
35	del(19)	del(19)	del(19)+T790M	del(19)	-	-	-	-
36	del(19)	del(19)	del(19)+T790M	WT	-	-	-	-
37	del(19)	WT	del(19)+T790M	del(19)	del(19)	del(19)+T790M	del(19)+T790M	del(19)+T790M
38	del(19)	del(19)+T790M	-	-	-	-	-	-

WT: Wild Type; -: not done.
